# The Role of Selected Flavonols in Tumor Necrosis Factor-Related Apoptosis-Inducing Ligand Receptor–1 (TRAIL-R1) Expression on Activated RAW 264.7 Macrophages

**DOI:** 10.3390/molecules20010900

**Published:** 2015-01-08

**Authors:** Monika Warat, Tadeusz Sadowski, Ewelina Szliszka, Wojciech Król, Zenon P. Czuba

**Affiliations:** 1 School of Medicine with the Division of Dentistry in Zabrze, Medical University of Silesia in Katowice, Chair and Department of Microbiology and Immunology, Jordana 19, 41-808 Zabrze, Poland; E-Mails: monika@warat.pl (M.W.); eszliszka@sum.edu.pl (E.S.); wkrol@sum.edu.pl (W.K.); 2 School of Public Health in Bytom, Medical University of Silesia in Katowice, Toxicology and Drug Addiction Division, Communal Department of Hygiene and Sanitary Supervision, Medyków 18, 40-752 Katowice, Poland; E-Mail: tadsad@poczta.onet.pl

**Keywords:** galangin, kaempferol, kaempferide, quercetin, Tumor Necrosis Factor-Related Apoptosis-Inducing Ligand)-Receptor (TRAIL-R1) expression, RAW264.7 macrophages

## Abstract

Tumor Necrosis Factor-Related Apoptosis-Inducing Ligand Receptors (TRAIL-R) are an important factor of apoptosis in cancer cells. There are no data about the effect of flavonols on the receptor expression on a surface of macrophage like cells. In this study, the expression level of TRAIL-R1 on murine RAW264.7 macrophages in the presence of selected flavonols: galangin, kaempferol, kaempferide and quercetin, which differ from their phenyl ring substituents, were studied. The expression of TRAIL-R1 death receptors on non-stimulated and lipopolysaccharide (LPS)-stimulated macrophages was determined using flow cytometry. The results suggested that compounds being tested can modulate TRAIL-R1 expression and can enhance TRAIL-mediated apoptosis.

## 1. Introduction

Tumor necrosis factor (TNF)-related apoptosis-inducing ligand (TRAIL/Apo2L) was discovered independently by two groups [1,2]; both groups reported sequence homology with the extracellular domain of CD95 ligand (Fas-L) and TNF. TRAIL is expressed as a type II transmembrane protein. To date, human TRAIL has been shown to interact with five receptors, including the death receptors TRAIL-R1/DR4/TNFRSF10A and TRAIL-R2/DR5/KILLER/TNFRSF10B and the decoy receptors TRAIL-R3/DcR1/TNFSRF10C and TRAIL-R4/DcR2/TNFSRF10D. In addition to these four membrane-bound receptors, osteoprotegerin (OPG, an inhibitor of RANK ligand), as a soluble receptor, is able to bind TRAIL. Death receptors with their cytoplasmic death domains are able to induce an apoptotic pathway. By contrast, death receptors without an intracellular death domain (DcR1) or with non-functional death domain (DcR2) are unable to induce a cell apoptosis. Like other members of the Tumor Necrosis Factor Receptor Superfamily (TNFRSF) TRAIL receptors are also characterized by a series of cysteine-rich repeats within their extracellular domains [3,4,5,6,7,8,9,10,11,12,13]. In the mouse, only death receptors resembling human TRAIL-R1 and TRAIL-R2 exist, two DcRs that are only distantly sequence-related to human DcR1 and DcR2 and one close homolog to OPG. The human and murine TRAIL protein shares 65% sequence identity [14,15]. 

**Figure 1:**
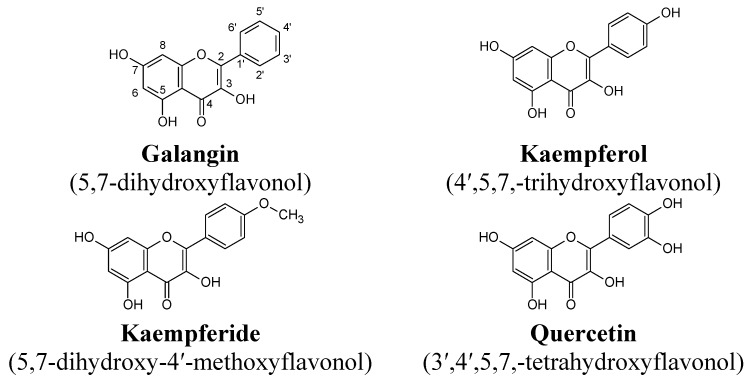
Chemical structures of the tested flavonols.

Our previously study indicated that RAW264.7 macrophages express TRAIL-R1, but not TRAIL-R2 receptors on their surfaces. Moreover, lipopolysaccharide (LPS 200 ng/mL) was found to significantly increase TRAIL-R1 death receptor expression, and additionally had no effect of TRAIL-R2 death receptor expression levels [16]. Now we have tested for the first time the expression of TRAIL-R1 and TRAIL-R2 on non-activated and LPS-stimulated RAW264.7 macrophages under influence of flavonols. Four substances belonging to flavonoids: galangin, kaempferol, kaempferide and quercetin (Figure 1), which differ in their substituents in phenyl ring, were used in the assays due to the variety of biological activities of polyphenol compounds (e.g., cancer prevention).

## 2. Results and Discussion

Viability of RAW264.7 macrophages in the presence of LPS (200 ng/mL) and/or flavonols (20 μM) was measured by MTT (3-(4,5-dimethyl-2-thiazyl)-2,5-diphenyl-2*H-*tetrazolium bromide) assay and LDH (lactate dehydrogenase) test. The results obtained showed that LPS and tested flavonols at the tested concentration had no significantly effect on the viability and was not toxic to macrophages. There were not differences between control values and tested samples examined by LDH assay (data not shown). The previously obtained results showed that flavonols had no effect on macrophage viability at the tested concentration. At concentration of 50 μM some flavonols had cytotoxic effects on RAW264.7 cells, that excluded the increase concentration of tested compounds, and we used one, lower concentration for all compounds (data not shown). Furthermore, for tested kaempferide, 20 μM was the highest concentration that can be obtained due to the limited solubility of this compound in the culture medium at the maximum concentration of 0.1% DMSO [17,18,19].

The macrophage cells have surface signaling molecules and receptors that determine their role in immunological reactions and mediate their interactions with natural and altered-self components of the host as well as a range of microorganisms [20,21,22].

**Figure 2:**
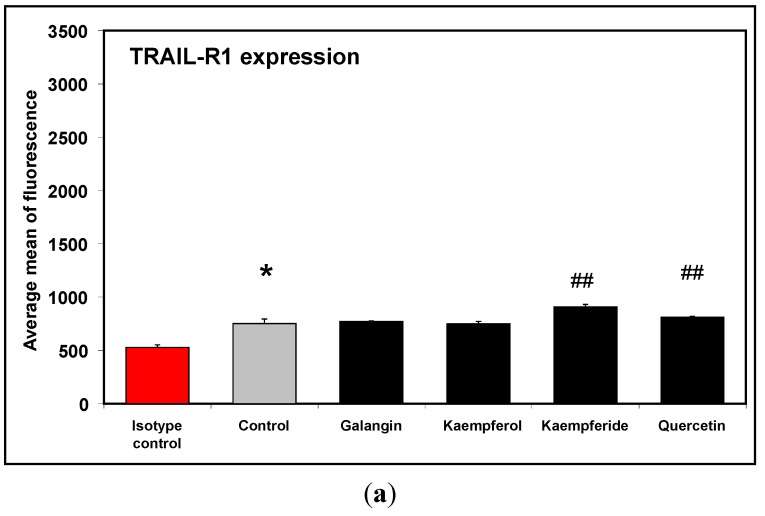

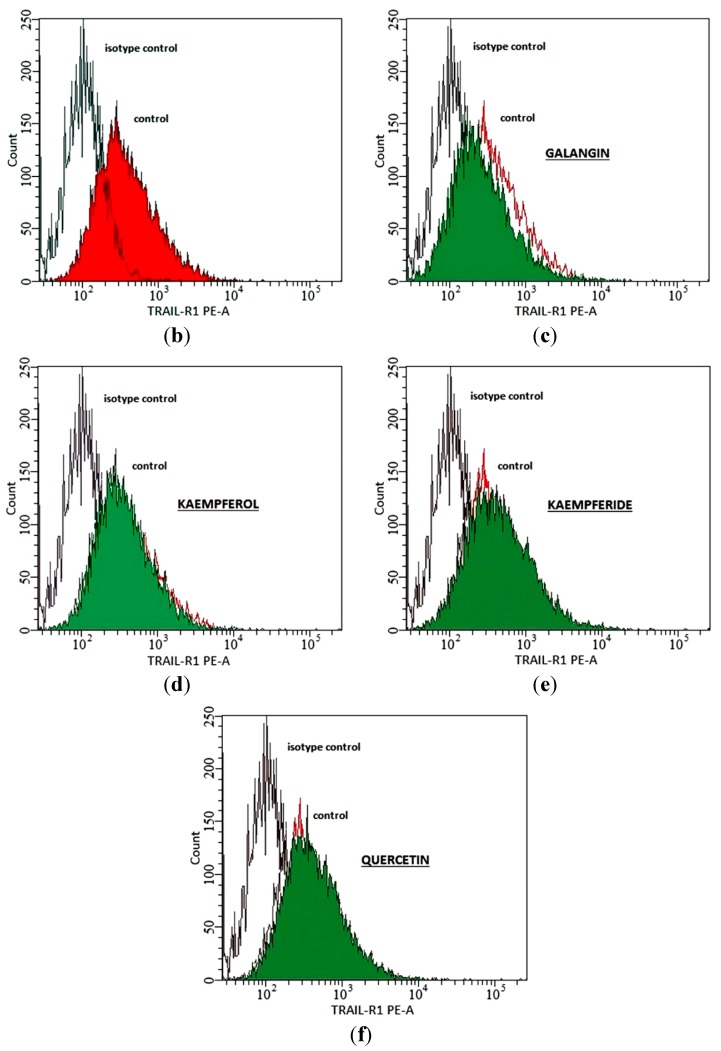
Effects of flavonols on TRAIL-R1 expression in RAW264.7 cells (**a**). Cells were incubated for 24 h alone (**b**), with galangin (**c**), kaempferol (**d**), kaempferide (**e**) and quercetin (**f**) at a concentration of 20 μM. The surface expression of TRAIL-R1 death receptors was measured by flow cytometric analysis. The values represent mean ± SD of three independent experiment, performed in duplicate *n* = 6. * *p* < 0.05 compared to isotype control. **^##^** *p* < 0.01 compared to control.

The level of TRAIL-R1 and TRAIL-R2 expression on the surface of RAW264.7 was estimated on native and stimulated LPS cells. Our results demonstrate that the macrophage cell line RAW264.7 have only the TRAIL-R1 receptors and LPS can enhance their expression. Next, the surface expression of TRAIL-R1 and TRAIL-R2 after a 24 h incubation of macrophage cells with flavonols at the concentration of 20 μM by flow cytometry was measured. After treatment with galangin and kaempferol, the expression level of TRAIL-R1 death receptor in macrophage cells did not change compared to control. However quercetin and kaempferide significantly increased expression level of the TRAIL-R1 receptor, by a mean of 7.7% and 19.92% respectively (Figure 2). Additionally none of the tested flavonols had an effect on TRAIL-R2 expression in non-activated cells (data not shown).

**Figure 3:**
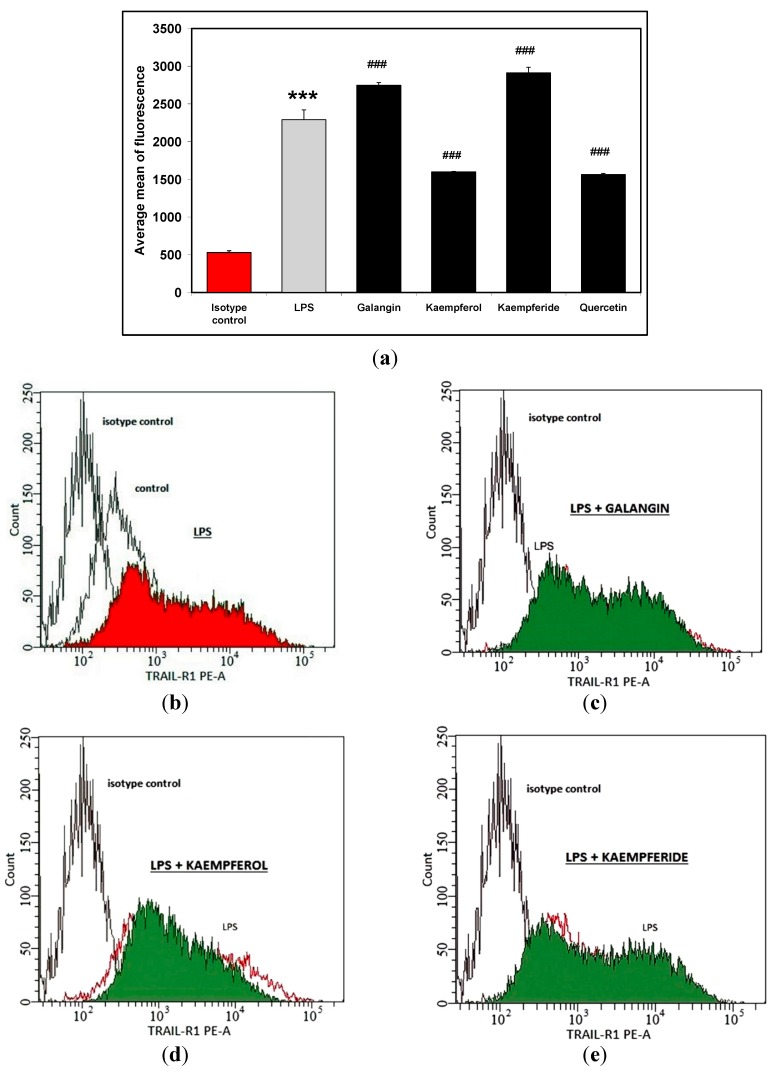

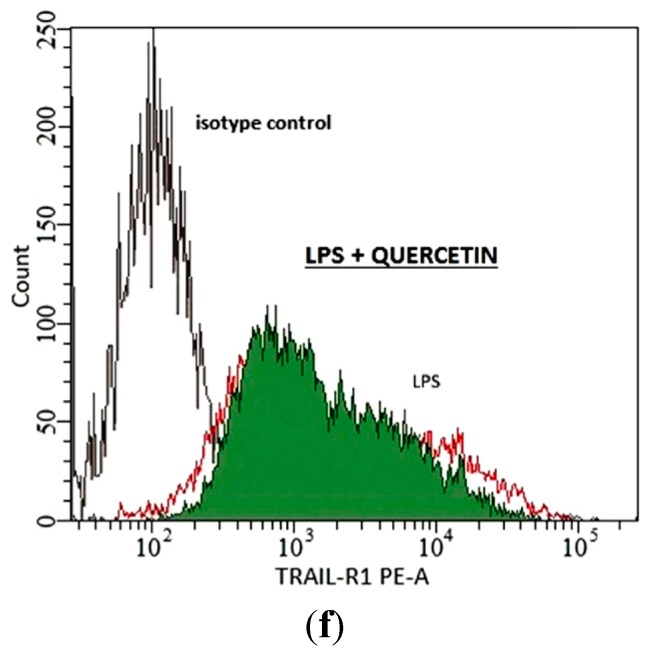
Effects of flavonols on TRAIL-R1 expression in RAW264.7 cells (**a**); Cells were incubated with LPS (200 ng/mL) (**b**); with LPS and galangin (**c**); with LPS and kaempferol (**d**), with LPS and kaempferide (**e**) and with LPS and quercetin (**f**) at concentration of 20 μM. The surface expression of TRAIL-R1 death receptor was measured by flow cytometric analysis. The values represent mean ± SD of three independent experiments, performed in duplicate *n* = 6. *** *p* < 0.001 compared to isotype control. **^###^** *p* < 0.001 compared to LPS-stimulated cells.

Next TRAIL-R1 and TRAIL-R2 expression level on LPS-stimulated macrophages was determined. The results showed that two of tested flavonols significantly increased levels of TRAIL-R1 expression on LPS-activated cells by a mean of 20% (for galangin), and 27.1% (for kaempferide). Moreover, both kaempferol and quercetin reduced TRAIL-R1 expression level by mean of 30% (Figure 3). The combination of LPS and tested flavonols did not induce TRAIL-R2 expression in studied cells (data not shown).

TRAIL selectively induces apoptosis (programmed cell death) in cancer and transformed cells, with no toxicity against normal cells and tissues. Importantly there also appears to be a complete lack of apparent toxicity, specifically hepatotoxicity, when TRAIL is used *in vivo* [1,23,24]. The expression level of TRAIL death receptors appears to be the most important factor of TRAIL-mediated apoptosis. However several experimental studies suggested that polyphenol compounds enhanced TRAIL-induced apoptosis by various molecular targets.

**Figure 4:**
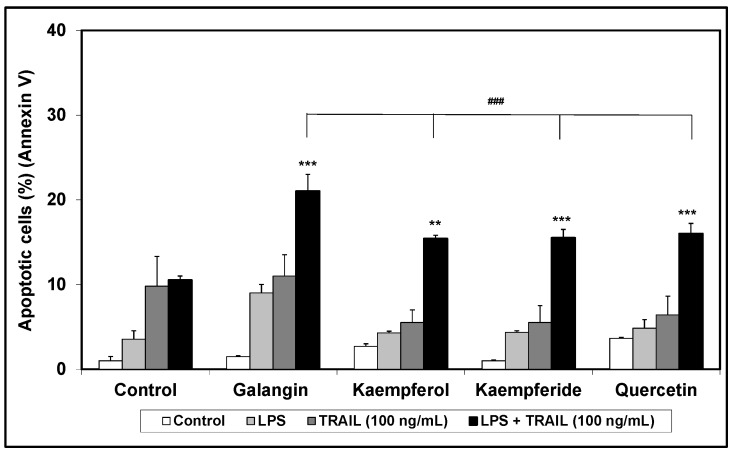
Apoptotic activity of TRAIL in combination with flavonols in RAW264.7 cells. The macrophages were incubated for 24 h with TRAIL (100 ng/mL) and flavonols (20 μM). Detection of apoptotic cell death by annexin V-FITC staining using flow cytometry. ** *p* < 0.01; *** *p <* 0.001 compared to control and **^###^** *p* < 0.001 compared to galangin sample.

In this experimental part we examined the cytotoxic effect of TRAIL (concentration: 20 ng/mL, 50 ng/mL and 100 ng/mL) alone and in combination with the tested flavonols on RAW264.7 macrophages, using flow cytometry. Tested flavonols did not have cytotoxic effects on non-stimulated and LPS-stimulated macrophages compared to controls (Figure 4). 

Only TRAIL at a concentration of 100 ng/mL induced a minor apoptotic effect in activated cells (10.5% ± 0.5%). Moreover, galangin in combination with TRAIL (100 ng/mL) increased the percentage of apoptotic cells by mean of 11%. Kaempferol, kaempferide and quercetin enhanced TRAIL-induced apoptosis by mean of 5.5%, 5.5% and 6.4% respectively. The apoptotic effect of TRAIL in combination with flavonols is shown in Figure 4. There are no papers describing death receptor expression on murine RAW264.7 macrophages under influence of flavonols. Chen *et al.* [25] demonstrated that quercetin significantly enhances tumor necrosis factor-related apoptosis-inducing ligand (TRAIL)-induced cytotoxicity in non-small cell lung cancer (human NSCLC cell line) cells. The authors showed that quercetin increased expression of TRAIL-R2 death receptor expression. Our previously study, performed on LNCaP cell line, confirmed the ability quercetin with TRAIL to induce cell apoptosis [26]. Jung *et al.* [27] reported that quercetin increased expression of TRAIL-R2 in prostate cancer cells–DU-145 human cell line. Additionally the authors showed that quercetin increased DR5 protein levels dose-dependently in DU-145, PC3, and LNCaP cells, which indicates that upregulation of DR5 may be a common response of prostate cells to quercetin exposure. Enhanced TRAIL-induced apoptosis by quercetin-induced DR5 upregulation is associated with increased activation of the caspase pathway. Yoshida *et al.* [28] demonstrated for the first time that the combined treatment with kaempferol and TRAIL drastically induced apoptosis in human colon cancer SW480 cells, compared to single treatments. Kaempferol markedly upregulated TRAIL receptors, DR5 and DR4. The authors showed that apoptosis, induced by the combination of kaempferol and TRAIL, can be efficiently blocked by DR5 siRNA but not by DR4 siRNA, indicating that DR5 upregulation by kaempferol helps to enhance TRAIL actions. Numerous dietary polyphenols have been reported to upregulate expression of death receptors—TRAIL-R1 and/or TRAIL-R2 in malignant human tumor cells—apigenin [29,30], luteolin [31], baicalein [32], epigallocatechin-3-gallate [33], silibilin [34] and resveratrol [35]. Our previously study, performed on RAW264.7 cell line, indicated that chrysin and apigenin significantly reduced the expression level of TRAIl-R1 both on non-stimulated and LPS-stimulated macrophages, and acacetin reduced TRAIL-R1 expression on LPS-stimulated cells [16].

In this study for the first time we report the influence of flavonols on TRAIL-R death receptors expression level on RAW264.7 macrophages. The results from this study suggest that compounds being tested can modulate TRAIL-R1 surface expression. This ability correlates with their structure. The most characteristic feature in the structure of the flavonols is the presence of a hydroxyl group in position 3 in the phenyl ring. A methoxyl group in the 4′ position in the kaempferide structure resulted in the strongest receptor expression stimulation on LPS-stimulated macrophages. Lack of a substituent in the 4′ position in the galangin structure determined weaker stimulation of TRAIL-R1 receptor expression compared to kaempferide. However a hydroxyl group in that position in the kaempferol structure and hydroxyl groups in 3′ and 4′ positions in the phenyl ring structure in quercetin resulted in inhibition of TRAIL-R1 receptor expression on LPS-stimulated macrophages and enhanced apoptosis induced with TRAIL. This mechanism is not yet clear and probably is the sum of contributions. The studied cells activated with LPS are able to generate many reactive substances. Among others there are TNF-α, and reactive forms of oxygen and nitrogen. Kaempferol and quercetin inhibit this activity. Other studied cancer cell lines (not macrophage like cells) did not generate a lot amount of reactive forms of oxygen and nitrogen compared to stimulated macrophage cell lines. Quercetin and kaempferol show the strongest antioxidant activity among the tested flavonols and can inhibit the luminol-enhanced chemiluminescence of neutrophils. Flavones (without a hydroxyl group in the 3 position) and similar phenyl ring structures decreased the chemiluminescence of neutrophils to a smaller degree [36,37]. Antioxidant activity, inhibition of enzymes and penetration of these compounds into cells might be important in the induction of apoptosis in the tested cells.

## 3. Experimental Section 

### 3.1. Cell Culture

Murine macrophage cell line RAW264.7 (established from a tumor induced by Abelson murine leukemia virus) was obtained from the ATCC (American Type Culture Collection, Manassas, VA, USA). The cells were cultured in Dulbecco’s modified Eagle’s medium (DMEM) supplemented with 10% heat inactivated fetal bovine serum (FBS, 10%), 4 mM l-glutamine, 100 U/mL penicillin, and 100 μg/mL streptomycin and maintained in monolayer cultures at the temperature 37 °C and atmosphere containing 5% CO_2_. Reagents for cell culture were purchased from ATCC. RAW264.7 were seeded at a density of 1 × 10^6^ cells/mL (2 × 10^5^ cells/well) in 96-well plates with or without LPS (200 ng/mL) and with or without flavonols for 24 h [16,38,39].

### 3.2. Flavonols

Tested flavonols were obtained from Rotichem HPLC, Carl Roth KG-D75, Karlsruhe, Germany. The compounds were dissolved in dimethyl sulphoxide (DMSO) to obtain the working concentration solutions. Lipopolysaccharide (LPS *E. coli* O:111:B4) was purchased from Sigma Chemical Company, (St. Louis, MO, USA).

### 3.3. Cell Viability

The viability was assessed by a colorimetric MTT (3-(4,5-dimethyl-2-thiazyl)-2,5-diphenyl-2*H-*tetrazolium bromide) assay, as previously described [16,38,40]. Briefly, the RAW264.7 cells (1 × 10^6^ cells/mL) were seeded 3 h before the experiments in a 96-well plate, washed and treated with flavonols (20 μM) with or without LPS (200 ng/mL) for 24 h. Final volume was 200 μL. Next the medium was removed, and MTT solutions (20 μL, 5 mg/mL; Sigma Chemical Company) were added to each well for 2 h. The resulting formazan crystals were dissolved in DMSO (100%). Controls included native cells and medium alone. The absorbance of each well was measured at 550 nm using an ELx 800 microplate reader (Bio-Tek Instruments Inc., Winooski, VT, USA). The viability was calculated by the formula: percent of viable cells = (absorbance of experimental wells/absorbance of control wells) × 100%.

### 3.4. Lactate Dehydrogenase Release Assay

Lactate dehydrogenase (LDH) is a stable cytosolic enzyme released upon membrane damage in necrotic cells. LDH activity was measured using a commercial Cytotoxicity Detection kit (Roche Diagnostics GmbH, Mannheim, Germany). The RAW264.7 cells (1 × 10^6^ cells/mL) were treated with flavonols (20 μM) with or without LPS for 24 h. The activity of LDH leaked from cells to culture supernatants was measured by an enzymatic assay, base of the conversion of a tetrazolium salt into a red formazan product. The control with maximal release of LDH from cells was obtained after treating the cells with 1% Triton X-100 (Sigma Chemical Company) for 10 min at room temperature. Next the absorbance of each well at 490 nm was measured using an ELx 800 microplate reader. The percentage of cytotoxic cells was calculated using the formula: (sample value/maximal release) × 100% [16,38,40].

### 3.5. Flow Cytometric Analysis of Death Receptor Expression on the RAW264.7 Cells

The cell surface expression of TRAIL-R1 and TRAIL-R2 death receptors was detected by flow cytometry (LSR II Flow Cytometer; BD Biosciences, San Jose, CA, USA). RAW264.7 macrophages (2.5 × 10^5^ cells/mL) were seeded in 24-well plates for 24 h and exposed to flavonols (20 μM) for 24 h. Cells were then harvested mechanically, washed twice with PBS (phosphate buffered saline) and resuspended in PBS containing 0.5% bovine serum albumin. Cells were incubated with 10 μL phycoerythrin-conjugated anti-TRAIL-R1 or anti-TRAIL-R2 monoclonal antibodies (R&D Systems, Minneapolis, MN, USA) at 4 °C for 60 min. After staining, the cells were washed with PBS and analysed using flow cytometry. The number of counted cells was 10^4^. The control sample (isotype control for TRAIL-R1) consisted of the cells only treated with phycoerythrin-labelled mouse IgG1 or (isotype control for TRAIL-R2) phycoerythrin-labelled mouse IgG2B (R&D Systems). Final volume of samples was 400 μL [16,26,41].

### 3.6. Detection of Apoptosis by Flow Cytometry

Apoptosis was measured using flow cytometry to quantify the levels of phosphatidylserine (PS) on the outer membrane of apoptotic cells. The PS on the outer surface of the cytoplasmic membrane was labelled by Annexin V (compound with a high affinity for PS) conjugated with FITC. The Annexin V assay was performed using the Apoptotest™-FITC Kit (Dako, Glostrup, Denmark). RAW264.7 macrophages (2 × 10^5^ cells/mL) were seeded in 24-well plates for 24 h and then exposed to flavonols (20 μM) and/or TRAIL (20–100 ng/mL) for 24 h. After this time RAW264.7 cells were washed twice with PBS and resuspended in binding buffer (0.5 mL). The cell suspension was then incubated with Annexin V-FITC (5 μL) and propidium iodide (PI, 5 μL) for 10 min at room temperature in the dark. The population of Annexin V-positive cells was evaluated by flow cytometry (LSR II Flow Cytometer; BD Biosciences). The number of counted cells was 10^4^ [16,26,41].

## 4. Conclusions

Our study indicated that tested flavonols can modulate TRAIL-R1 death receptors on RAW264.7 macrophages. Galangin enhanced TRAIL-mediated apoptosis of RAW264.7 macrophages due to increase TRAIL-R1 expression. In spite of inhibiting TRAIL-R1 expression level on macrophages kaempferol, kaempferide and quercetin enhanced TRAIL-mediated apoptosis due to various molecular targets.
